# Performance of Microarray and Liquid Based Capture Methods for Target Enrichment for Massively Parallel Sequencing and SNP Discovery

**DOI:** 10.1371/journal.pone.0016486

**Published:** 2011-02-09

**Authors:** Anna Kiialainen, Olof Karlberg, Annika Ahlford, Snaevar Sigurdsson, Kerstin Lindblad-Toh, Ann-Christine Syvänen

**Affiliations:** 1 Department of Medical Sciences, Uppsala University, Uppsala, Sweden; 2 Department of Medical Biochemistry and Microbiology, Uppsala University, Uppsala, Sweden; 3 Broad Institute of Harvard and Massachusetts Institute of Technology (MIT), Cambridge, Massachusetts, United States of America; Duke-National University of Singapore Graduate Medical School, Singapore

## Abstract

Targeted sequencing is a cost-efficient way to obtain answers to biological questions in many projects, but the choice of the enrichment method to use can be difficult. In this study we compared two hybridization methods for target enrichment for massively parallel sequencing and single nucleotide polymorphism (SNP) discovery, namely Nimblegen sequence capture arrays and the SureSelect liquid-based hybrid capture system. We prepared sequencing libraries from three HapMap samples using both methods, sequenced the libraries on the Illumina Genome Analyzer, mapped the sequencing reads back to the genome, and called variants in the sequences. 74–75% of the sequence reads originated from the targeted region in the SureSelect libraries and 41–67% in the Nimblegen libraries. We could sequence up to 99.9% and 99.5% of the regions targeted by capture probes from the SureSelect libraries and from the Nimblegen libraries, respectively. The Nimblegen probes covered 0.6 Mb more of the original 3.1 Mb target region than the SureSelect probes. In each sample, we called more SNPs and detected more novel SNPs from the libraries that were prepared using the Nimblegen method. Thus the Nimblegen method gave better results when judged by the number of SNPs called, but this came at the cost of more over-sampling.

## Introduction

Recent development of massively parallel sequencing technologies has remarkably increased the throughput and brought down the costs of sequencing [1], [2]. As a result, it is currently feasible to sequence even whole human genomes in a time frame of months, or even weeks, rather than years [3], [4], [5], [6], [7]. Though possible, whole genome sequencing is still more laborious, time consuming, and expensive than sequencing sub-regions of the genome. Data analysis is also simplified by concentrating on specific sub-regions of the genome. Thus in many projects, targeted sequencing of specific regions of interest in a large number of samples continues to be much more cost-effective in providing answers to biological questions than sequencing the whole genomes of fewer individuals.

Targeted sequencing of all human exons has already led to the discovery of a growing number of genes behind inherited disorders [8], [9], [10], [11], [12], [13], brain malformations [14], and cancer metastasis [15]. In addition to selecting regions of genomic DNA, target enrichment combined with massively parallel sequencing has a number of other applications. It has been used for sequencing RNA (cDNA) to study subsets of the transcriptome [16] as well as studying RNA editing [17], allele specific gene expression [18], and DNA methylation [19].

Several approaches have been developed to select the regions of interest for sequencing from the genome [20]. Polymerase chain reaction (PCR) has traditionally been used to enrich the genomic fragments for Sanger sequencing. PCR selection of 144 target regions in pooled DNA samples has been used successfully in combination with massively parallel sequencing to discover new SNPs associated with type 1 diabetes [21], but in addition to becoming laborious and expensive when used for the enrichment of larger genomic regions, even the combination of multiplex and long-range PCR does not match the current throughput of the massively parallel sequencing technologies. Thus a lot of effort has recently been put into the development of more efficient enrichment methods.

Hybrid selection using cDNA [22] and bacterial artificial chromosomes (BACs) [23] have been used for selecting specific genomic regions even before the development of massively parallel sequencing technologies. DNA capture using concatenated PCR products immobilized on filters as subgenomic traps [24] and multicycle hybridization on microfluidic chips [25] were recently described, but these methods are only scalable for enriching regions of up to one megabase in size. Microdroplet-based PCR enrichment has been successfully used for targeting 435 exons of 47 genes [26]. The Gene Collector method [27], selector probes [28], [29], and padlock probes/molecular inversion probes (MIPs) produced by array-released oligo libraries [30], [31] are enrichment techniques, in which the target DNA is circularized by a hybrid selection step followed by a ligation step and then amplified. In array based hybridization capture methods, hundreds of thousands of oligonucleotides complementary to the region of interest are synthesized on high-density DNA microarrays [32], [33], [34], [35]. In the solution based hybridization capture method, array-released oligo libraries are converted into biotinylated *in vitro* transcribed RNA probes, which are used to capture the regions of interest [36], [37]. These methods are suitable for the enrichment of large genomic regions, including whole exomes.

Our goal was to choose the optimal enrichment method for variant discovery in continuous custom-selected target regions. The choice of the Nimblegen array and SureSelect solution based hybridization capture methods for the comparison presented here was based on their throughput and general availability. In order to evaluate these methods, we prepared sequencing libraries from three HapMap samples using both methods for the same target region and sequenced the libraries using a Genome Analyzer IIx instrument (Illumina). We called sequence variants and compared the results to the existing data for the HapMap samples as well as to data in publicly available databases and validated the findings by Sanger sequencing.

## Materials and Methods

### Samples

DNA samples from three immortalized lymphoblastoid cell lines, NA10860, NA11992, and NA11993, were purchased from the Coriell Cell repository (Camden, NJ).

### Nimblegen array hybridization

The protocol was modified from previous descriptions [33], [34]. Fifteen µg of genomic DNA in 100 µl TE-buffer was first sonicated using cycles of 30 s on and 30 s off for a total of 15 min with high power in a water bath sonicator (Bioruptor, Diagenode) to obtain fragments of <800 bp. The fragmentation efficiency was evaluated by capillary electrophoresis on DNA1000 chips (Agilent 2100 Bioanalyzer, Agilent). Sequencing adapters were ligated on the DNA fragments according to manufacturer's instructions (Illumina). Three parallel reactions were done for each sample. To obtain blunt ended fragments, 45 µl of H_2_O, 10 µl of T4 DNA ligase buffer with 10 mM ATP (New England Biolabs (NEB)), 4 µl 10 mM dNTP mix (Invitrogen), 5 µl T4 DNA polymerase (NEB), 1 µl Klenow DNA polymerase (NEB), and 4 µl T4 polynucleotide kinase (NEB) were added to 5 µg of fragmented DNA in 30 µl EB (Qiagen) and incubated for 30 min at 20°C. The reaction product was purified with the QIAquick PCR purification kit (Qiagen) and the DNA was eluted into 32 µl of EB. To add A bases to the 3′ ends of the DNA fragments, 5 µl of NEB2 buffer (NEB), 10 µl 1 mM dATP, and 3 µl Klenow exo- (NEB) were added to the DNA and incubated for 30 min at 37°C. The reaction product was purified with the MinElute PCR purification kit (Qiagen) and eluted into 10 µl EB. Adapters were ligated to the ends of the DNA fragments by adding 25 µl Quick ligase buffer (NEB), 10 µl adapter oligo mix (Illumina), and 5 µl Quick ligase (NEB) and incubating for 15 min at 20°C. The reaction product was purified with the QIAquick PCR purification kit (Qiagen) and eluted into 30 µl EB. The three reaction products were pooled and the quantity of the adapter ligated DNA was measured with a Nanodrop ND-1000 spectrophotometer (Nanodrop Technologies). Fifty µg of human Cot-1 DNA (Invitrogen) and 1 µmol of each blocking oligo (complementary to the sequencing adapters; IDT) were added to the adapter ligated DNA. The samples were dried in a DNA120 SpeedVac concentrator (Thermo Electron), rehydrated with 4.8 µl H_2_O and incubated for 10 min at 70°C. Eight µl of 2xSC hybridization buffer (Roche Nimblegen) and 3.2 µl SC hybridization component A (Roche Nimblegen) were added to the rehydrated DNA. The DNA was denatured for 10 min at 95°C and then kept at 42°C until it was loaded onto the arrays. Fifteen µl of the sample was loaded on the 385K feature custom designed sequence capture arrays (Roche Nimblegen) and incubated for 65 hours with mixing at 42°C. After the hybridization, the slide was washed once in wash buffer II (Roche Nimblegen) at room temperature (RT), twice with stringent wash buffer (Roche Nimblegen) for 5 min at 47.5°C, once with wash buffer I (Roche Nimblegen) for 2 min with 1 inversion/s at RT, once with wash buffer II (Roche Nimblegen) for 1 min with 1 inversion/s at RT, and once with wash buffer III (Roche Nimblegen) with 10 inversions at RT. DNA was eluted from the arrays, twice for 5 min at 95°C, and once without incubation with 400 µl H_2_O. All three eluates were collected in the same tube and the sample dried in a DNA120 SpeedVac concentrator (Thermo Electron). The sample was rehydrated with 20 µl of H_2_O. Three µl of the rehydrated elution product was used for the subsequent PCR with adapter-primers. The PCR reaction mixture also contained 1 µl primer 1.1 (Illumina), 1 µl primer 2.1 (Illumina), 25 µl Phusion master mix (Finnzymes), and 20 µl H_2_O. The PCR was performed as follows: 30 s at 98°C, 18 cycles of: 10 s at 98°C, 30 s at 65°C, and 30 s at 72°C, then 5 min at 72°C. The PCR product was purified with the QIAquick PCR purification kit (Qiagen) and eluted into 30 µl EB. The quality of the sequencing libraries was verified by capillary electrophoresis (Bioanalyzer, Agilent).

### SureSelect hybridization

SureSelect sequencing libraries were prepared according to the manufacturer's instructions (Agilent). Three µg of genomic DNA in 100 µl TE-buffer was fragmented to a median size of 200 bp using the Covaris-S2 instrument (Covaris) with the following settings: duty cycle 10%, intensity 5, cycles per burst 200, and mode frequency sweeping for 180 s at 4°C. The fragmentation efficiency was evaluated by capillary electrophoresis on DNA1000 chips (Agilent). Sequencing adapters were ligated on the DNA fragments following the manufacturer's protocol (Illumina) as described above for the Nimblegen method. Adapter ligated DNA was run on a 2% TAE-agarose gel (BioRad) and the region of the gel containing fragments in the 200–300 bp range was excised. The DNA was purified from the gel using a Gel extraction kit (Qiagen) and eluted in 30 µl EB. The adapter ligated and size selected DNA was amplified by PCR. One µl of DNA, 22 µl H_2_O, 1 µl primer 1.1 (Illumina), 1 µl primer 2.1 (Illumina), and 25 µl Phusion master mix (Finnzymes) were amplified as follows: 30 s at 98°C, 14 cycles of: 10 s at 98°C, 30 s at 65°C, and 30 s at 72°C, then 5 min at 72°C. The reaction product was purified with the QIAquick PCR purification kit (Qiagen) and eluted into 50 µl EB. The quality of the PCR products was assessed by capillary electrophoresis (Bioanalyzer, Agilent). SureSelect hyb #1, #2, #3, and #4 reagents (Agilent) were mixed to prepare the hybridization buffer. The adapter ligated DNA fragments were concentrated in a DNA120 SpeedVac concentrator (Thermo Electron) to 500 ng in 3.4 µl. SureSelect block #1, #2, and #3 reagents (Agilent) were added to the 500 ng of DNA. The hybridization buffer and the DNA blocker mix were incubated for 5 min at 95°C and then for 10 min at 65°C in a thermal cycler (MJ Research). RNase block (Agilent) was added to the SureSelect oligo capture library (Agilent). The capture library was incubated for 2 min at 65°C. First the hybridization buffer, and then the DNA blocker mix were added to the capture library and the mixture was incubated for 24 hours at 65°C in a thermal cycler (MJ Research). Fifty µl of streptavidin coated Dynabeads M-280 (Invitrogen) were washed three times with 200 µl SureSelect binding buffer (Agilent) and resuspended in 200 µl of the binding buffer. The hybridization mixture was added to the bead suspension and incubated for 30 min at RT with mixing. The beads were washed with 500 µl SureSelect wash buffer #1 (Agilent) for 15 min at RT, and three times with 500 µl SureSelect wash buffer #2 (Agilent) for 10 min at 65°C. DNA was eluted with 50 µl SureSelect elution buffer (Agilent) for 10 min at RT. Fifty µl of SureSelect neutralization buffer (Agilent) was added to the eluted DNA. The reaction product was purified with the MinElute PCR purification kit (Qiagen) eluting in 15 µl EB. Two PCR reactions with 1 µl of the elution product, 1 µl primer 1.1 (Illumina), 1 µl primer 2.1 (Illumina), 25 µl Phusion master mix (Finnzymes), and 22 µl H_2_O were performed. The PCR conditions were as follows: 30 s at 98°C, 18 cycles of: 10 s at 98°C, 30 s at 65°C, and 30 s at 72°C, then 5 min at 72°C. The two reactions were combined and purified with the QIAquick PCR purification kit (Qiagen) and eluted into 30 µl EB. The quality of the sequencing libraries was verified by capillary electrophoresis (Bioanalyzer, Agilent) and the concentration was measured with a spectrophotometer (NanoDrop Technologies).

### Sequencing by the Genome Analyzer II*_X_*

Samples were sequenced on the Illumina Genome Analyzer II*_X_* (GAII*_X_*) following the manufacturer's instructions. A single read cluster generation kit v.2 (Illumina) was used to generate the clusters and a single read sequencing kit v.3 (Illumina) was used for sequencing. All samples were sequenced on a single lane of an Illumina flow cell in the same single read 36 cycle GA run.

### Data analysis

Image analysis and base calling of the raw sequencing data were performed using the Illumina data analysis pipeline v1.4. The sequences were aligned to the human genome reference (hg18, build 36) using BWA [38] with default parameters. The sequence reads that aligned to the targeted regions were then extracted from the BWA alignment using Samtools [39]. The extracted sequence reads were re-aligned to the targeted regions using Maq [40] with default parameters in order to perform SNP calling. In house scripts written in Perl were used for the downstream analyses of the obtained SNP lists as well as for making the coverage plots. The HapMap data used was the Feb 2009 phaseII+III data downloaded from the NCBI ftp-site (ftp.ncbi.nlm.nih.gov:/hapmap/genotypes/2009-02_phaseII+III/forward/non-redundant/). Plink [41] was used for inheritance analysis of novel SNPs.

### Sanger sequencing

PCR primers were designed using Primer3 (http://frodo.wi.mit.edu/primer3/) and Autoprimer (http://www.autoprimer.com, Beckman Coulter Inc.) and ordered from Integrated DNA Technology (IDT). Primer sequence information is available in Supplementary Table S1. Twenty ng of genomic DNA was amplified with 0.05 µM primers, 0.1 U/µL SmartTaq DNA polymerase (Naxo) and 80 µM dNTPs in 10 mM Tris–HCl, pH 8.8, 50 mM KCl, 5 mM MgCl_2_, 0.001% (w/v) gelatin in a reaction volume of 10 µl at 95°C for 7 min, 40 cycles of 94°C for 30 s, 55 or 60.5°C for 30 s and 72°C for 1 min. Amplification products were verified on 1.5% agarose gels and 7 µl of each product was incubated with 0.5 U/µL exonuclease I (Fermentas), and 0.1 U/µL shrimp alkaline phosphatase (GE Healthcare) in 10 µl of 48 mM Tris–HCl, pH 9.5, 7.6 mM MgCl_2_, at 37°C for 45 min, followed by inactivation of the enzymes at 85°C for 15 min. Purified PCR products were split into two reactions and sequenced on both strands (ABI3730XL, Applied Biosystems). The resulting traces were analyzed with the Sequence Scanner v.1.0 software (Applied Biosystems).

## Results

### Capture probe designs

To compare the performance of two generally available hybridization-based methods for capturing 56 genes, including exons, introns, and 5 kb of up- and downstream non-coding sequence for massively parallel sequencing, we ordered assays from two companies, Agilent and Roche Nimblegen. For each gene, the transcribed region was determined according to the longest annotation of the transcript in the consensus coding sequence regions (CCDS) database and the region was extended with 5 kb upstream and downstream of the transcript. Put together, these 56 gene regions of 13–234 kb in size made up a region of interest of 3.1 Mb in total size. The probe designs obtained from the companies were uploaded in the UCSC genome browser (http://genome.ucsc.edu/) and the probe positions were checked manually before acceptance. In the SureSelect method from Agilent, biotinylated RNA probes of 120 nucleotides in length are used to capture the desired DNA fragments from a pool of DNA fragments to which sequencing adapters have been ligated [36]. In the design that we ordered, each target position was covered by four different probes resulting in 30 bp offset between the probes. Agilent used RepeatMasker (http://www.repeatmasker.org/) to exclude repeated regions from the SureSelect design, which resulted in a probe set that targets 1.7 Mb (55%) of the original regions of interest. In the Nimblegen sequence capture method, the probes used are oligonucleotides synthesized on microarray slides. The length of the probes is adjusted to yield a uniform melting temperature (T_m_). In our design, the capture probes tile the region of interest with five base pair offset. Repeats were excluded from the design based on in-house repeat masking at Nimblegen [34], which resulted in a probe set that targets 2.4 Mb (78%) of the original target regions. 1.6 Mb (53%) of the original 3.1 Mb target region was targeted by both probe sets.

### Sequencing and alignment

We prepared sequencing libraries from three HapMap samples (NA10860, NA11992, and NA11993) using both the SureSelect target enrichment system from Agilent and the 385K sequence capture arrays from Roche Nimblegen using their standard protocols, and sequenced them in the same Genome Analyzer 36 bp single-read run. We obtained 10 to 13.4 million reads from the libraries prepared with the Nimblegen method and 18.3 to 19.1 million reads from the libraries prepared with the SureSelect method (Table 1). We were able to obtain more sequence reads from the SureSelect libraries since they had ∼14-fold higher concentrations than the Nimblegen array captured libraries, which made the quantification of the SureSelect libraries more accurate, which in turn resulted in optimal DNA amounts being deposited on the flow-cell for sequencing and higher cluster density.

**Table 1:** Sequencing and alignment statistics.

Sample	Number of reads (PF)	% of reads that align to genome	Number of reads that align to target	% of reads that align to target
NA10860 SureSelect (Downsampled)	18 325 890	89	13 803 067 (7603844)	75 (75)
NA10860 Nimblegen	10 093 998	85	5 363 593	53
NA11992 SureSelect (Downsampled)	18 785 249	90	13 818 498 (9293047)	74 (74)
NA11992 Nimblegen	12 634 643	83	5 209 663	41
NA11993 SureSelect (Downsampled)	19 148 344	89	14 200 810 (9972236)	74 (74)
NA11993 Nimblegen	13 444 317	88	8 985 107	67

PF  =  pass filter.

The error rate estimated by the Illumina GA-pipeline was approximately 0.3% based on an ELAND-alignment against the human genome reference sequence (hg18, build 36). Out of the sequence reads that passed the quality filter in the Illumina data analysis pipeline, 83 to 90% aligned to the human genome reference (Table 1). For the subsequent analysis, the sequence reads were re-aligned against the human genome reference using BWA [38]. BWA places the reads that align to multiple locations randomly to one of those locations. When the reads mapping to the target region were extracted from the alignment using Samtools [39], the reads that mapped to multiple locations, but had been randomly placed to our target region by BWA, were included. Between 41 and 75% of the quality filtered reads aligned to the target regions per sample (Table 1). In each sample, a larger proportion of the reads from the libraries prepared with the SureSelect method (74–75%) than from the ones prepared with the Nimblegen method (41–67%) aligned to the target regions (Table 1). The percentage of reads that aligned to the target region was more consistent between the SureSelect libraries. Thus the libraries prepared with the SureSelect method showed higher specificity and better reproducibility than the libraries prepared with the Nimblegen method.

**Figure 1:**
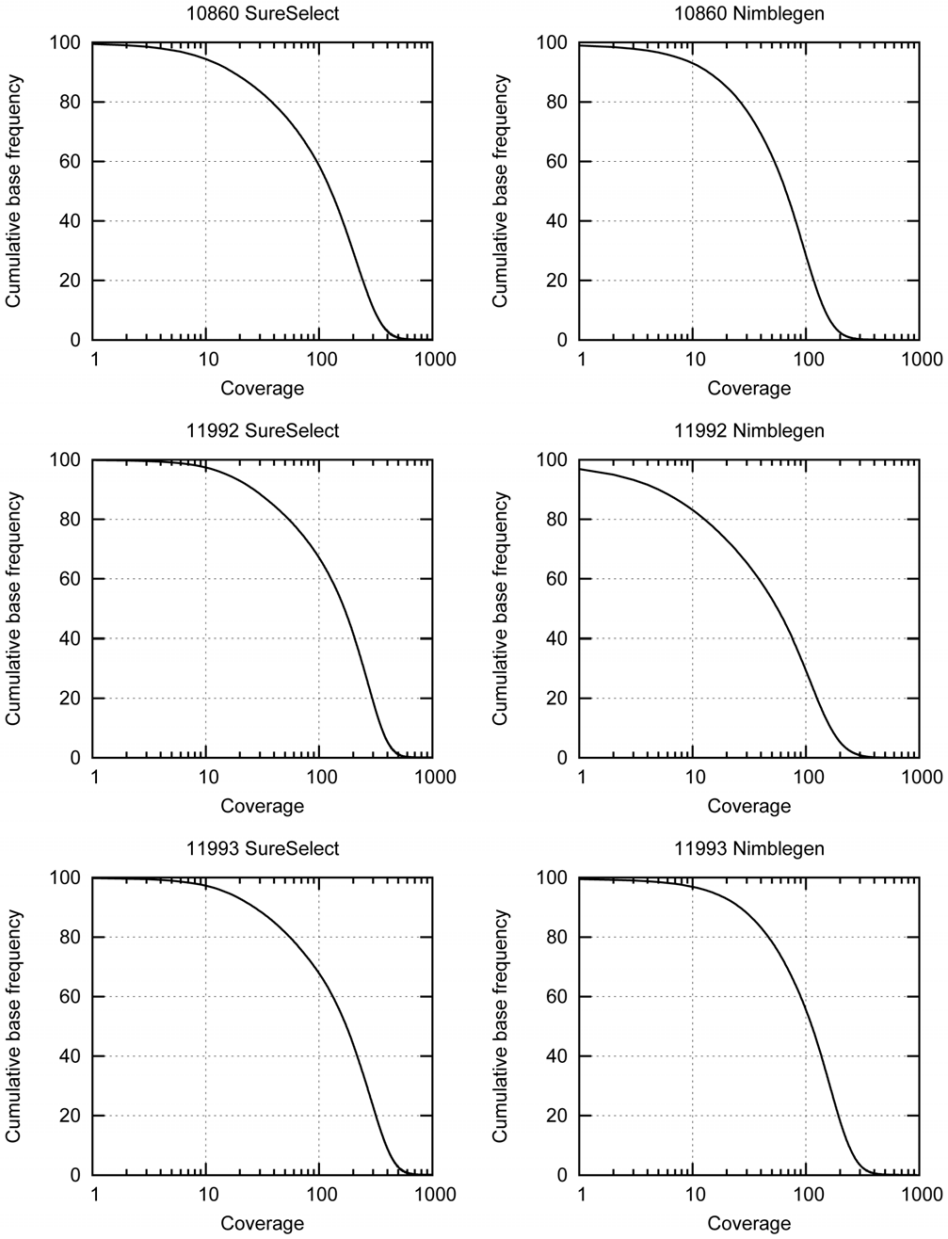
Coverage plots of the sequencing libraries prepared with SureSelect (left) and Nimblegen (right) methods. Cumulative base frequency is plotted against coverage. In the SureSelect libraries, 94–97% of the bases in the regions targeted by the probes are covered by at least ten reads. In the Nimblegen libraries the corresponding number is 83–97%.

**Figure 2:**
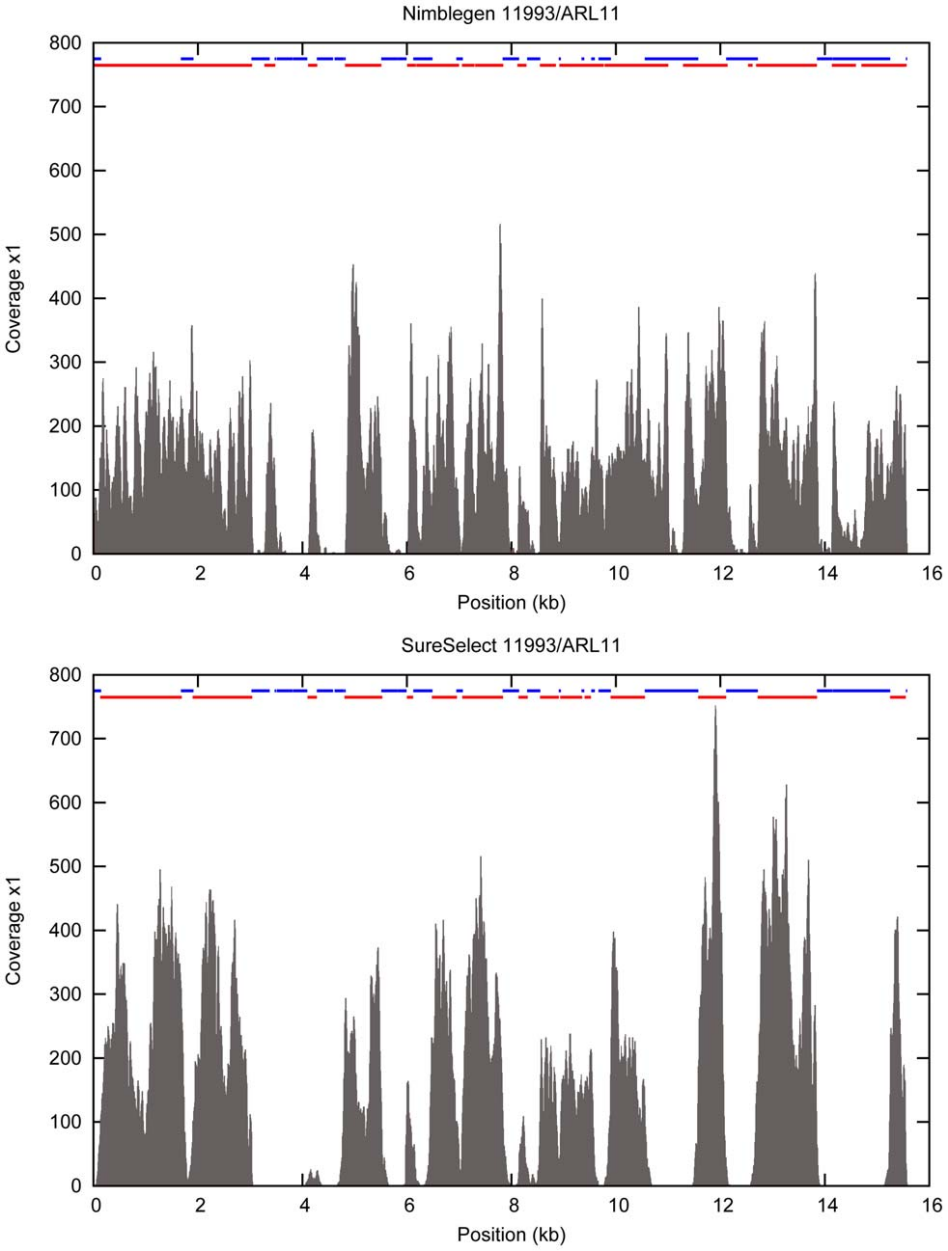
Coverage of a single gene. Per base coverage plotted along *ARL11* in the sample NA11993 in sequencing libraries prepared with Nimblegen (top) and SureSelect (bottom) methods. The bars at the top of the graph mark the regions that are targeted by probes in each design (red) and the repeat elements as determined by RepeatMasker (blue).

To avoid bias due to the higher number of sequence reads obtained from the SureSelect libraries in the subsequent analysis, we downsampled the reads in the SureSelect libraries to match the number of reads obtained from the Nimblegen libraries for each individual and repeated the alignments (Table 1). We then determined the completeness of coverage of the target region in the different libraries (Fig. 1). On average, we covered 99.7% of the regions targeted by the SureSelect probes (Table 2) at least once, which means that 1.7 Mb (55%) of the original target region was covered at least once by the 35 bp sequence reads. Of the regions targeted by the Nimblegen probes we were able to sequence 98.5% at least once. Thus we were able to sequence 2.3 Mb (76%) of the original target region at least once in the libraries prepared with the Nimblegen method. The proportion of the target region that was successfully sequenced at least once represents the proportion of the target region that we were able to capture i.e. the completeness of the library. Our results demonstrate that we obtain higher completeness for SureSelect libraries when we consider the regions targeted by the method specific probes. Nimblegen performs almost equally well when the regions targeted by both probe sets are considered and covers 0.6 Mb more of the original target region. We also looked at the completeness of coverage of the non-repeated parts of the target region, as these make up the biologically most relevant target. Nimblegen performed slightly better in these regions. 98.1% of the non-repeated part of the target was covered at least once in the libraries prepared with the Nimblegen method compared to 97.4% in the libraries prepared with the SureSelect method, on average (data not shown). However the results that we obtained when looking at the non-repeated part of the target region were very similar to the region targeted by both probe sets so they are not reported separately in the further analyses. An example of the coverage of one of the target genes as well as the positions of the probes and the repeat elements in both types of libraries is shown in Figure 2.

**Table 2:** Coverage of the probe targeted regions and regions targeted by both probe sets in each sample.

Sample	% probe targeted region/region targeted by both probe sets			Average sequencing depth
	>1X	>10X	>30X	
NA10860 SureSelect	99.5/99.5	94.4/94.4	83.6/83.4	86
NA10860 Nimblegen	99.0/99.5	93.0/94.8	77.1/80.7	61
NA11992 SureSelect	99.9/99.9	97.4/97.4	88.5/88.5	105
NA11992 Nimblegen	96.9/97.9	83.1/86.3	65.4/70.6	59
NA11993 SureSelect	99.8/99.8	97.3/97.3	88.7/88.7	113
NA11993 Nimblegen	99.5/99.8	96.9/98.0	88.1/90.6	101

**Figure 3:**
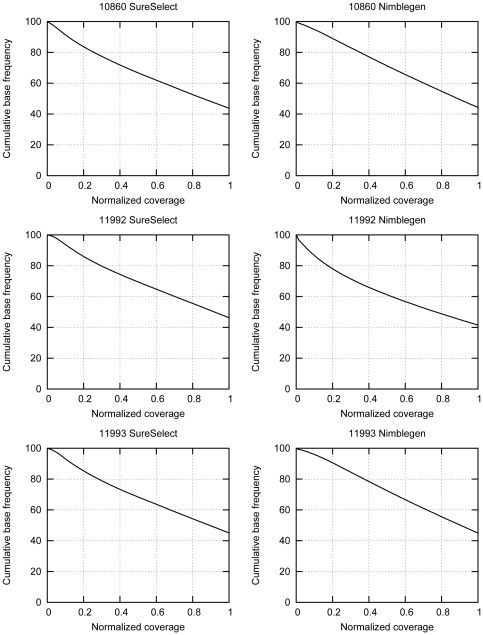
Normalized coverage of the sequencing libraries prepared with SureSelect (left) and Nimblegen (right) methods. To normalize the coverage, the absolute per base coverage was divided by the mean coverage. In the SureSelect libraries, 67–69% of the regions targeted by probes are covered by at least half of the average coverage. In the Nimblegen libraries 61–72% of the regions targeted by probes are covered by at least half of the average coverage.

We obtained average sequence depths of 59 to 101 for the Nimblegen libraries and 86 to 113 for the SureSelect libraries after downsampling (Table 2). To assess the uniformity of the representation of the targeted regions in the sequencing libraries in a sequencing depth independent way, we plotted the normalized coverage for each sample [36] (Fig. 3). We note that in the SureSelect libraries 67–69% of the targeted bases had at least half of the average coverage, whereas in the Nimblegen libraries this number was 61–72%. Thus, on average both methods performed equally well when judged by the uniformity of the coverage.

### SNP calling and comparison to HapMap positions

In the Nimblegen design, 94–96% of the genotyped SNP positions from HapMap for each of the three individual samples fell into the probe targeted regions, whereas 69–75% of the HapMap SNP positions in each individual sample were in the probe targeted regions in the Agilent design. 68–74% of the HapMap SNP positions were in the region targeted by both methods (Table 3). In total, we called between 2405 and 3371 SNPs per sample requiring a minimum coverage of ten (Table 4). For each DNA sample, the number of SNPs called was higher in the library that was prepared using the Nimblegen method than in the one prepared with the SureSelect method. In the region targeted by both methods, we called slightly more SNPs in the libraries that were prepared with the SureSelect method on average. To compare the base calls in our sequencing data to the genotypes from HapMap for the three samples that we sequenced, we made a list of SNP calls obtained using Maq and of base calls at all the positions that had been genotyped in HapMap and had a minimum of 10X sequencing coverage in our data for each sample. Since Maq did not call SNPs in the latter positions, we assumed that they were equal to the reference sequence. We then compared our sequencing based genotype list to the HapMap genotypes for each of the samples (Table 3). We were able to call 73–79% of the HapMap SNPs in the whole target region in the SureSelect libraries and 85–96% in the Nimblegen libraries. We correctly called 93–97% of the HapMap SNPs in the regions targeted by both methods in the SureSelect libraries and 85–94% in the Nimblegen libraries. We thus called a greater percentage of the HapMap SNPs in the regions targeted by both methods in the SureSelect libraries, but a greater percentage of the HapMap SNPs in the whole region of interest and a greater total number of SNPs in the Nimblegen libraries.

**Table 3:** HapMap SNPs in the targeted region for the three individuals sequenced.

	NA10860		NA11992		NA11993	
Number of SNPs	SureSelect	Nimblegen	SureSelect	Nimblegen	SureSelect	Nimblegen
In HapMap	977	977	1796	1796	1772	1772
Targeted by probes	673	923	1335	1722	1322	1701
Targeted by both probe sets	666	666	1323	1323	1310	1310
Covered by sequencing	710	884	1422	1519	1406	1708
Covered by probes and sequencing	652	870	1328	1498	1307	1678
Covered by both probe sets and seq	647	635	1317	1189	1297	1293
Sequencing agrees with HapMap	677	853	1378	1436	1375	1631
Targeted by both probe sets and seq agrees with HapMap	618	612	1279	1129	1271	1232

**Table 4:** SNPs in the targeted region and in the region covered by both probe sets.

	Total	HapMap	Annotated in Ensembl	In 1000 genomes	Novel
NA10860 SureSelect	2 405/2069	232/209	2245/1949	2227/1946	80/66
NA10860 Nimblegen	3 061/2089	310/209	2829/1953	2837/1937	131/87
NA11992 SureSelect	2 513/2161	645/598	2359/2051	2341/2031	67/53
NA11992 Nimblegen	2 598/1821	667/511	2411/1712	2409/1686	92/62
NA11993 SureSelect	2 702/2310	640/591	2453/2125	2483/2145	118/91
NA11993 Nimblegen	3 371/2221	758/556	3021/2031	3079/2049	181/98

96–98% of the SNPs called in the region targeted by both methods in the SureSelect libraries and 95–96% in the Nimblegen libraries were concordant with the HapMap genotypes (Table 3). The SNP calls, where the position of a SNP agreed, but where the actual base disagreed with the HapMap SNP data, were further inspected. Most discrepant calls between the HapMap data and the SNPs called in our sequences were heterozygous in the HapMap data, and homozygous according to our sequence data. We compared the HapMap genotypes and the genotypes obtained by sequencing from both types of libraries in all the positions where the sequencing call obtained with either method disagreed with the HapMap genotype in order to look for allelic drop outs. We saw more of what appeared to be allelic drop outs in the libraries prepared with the Nimblegen method in two of the samples and more allelic drop outs in the SureSelect library in the third sample. By manual inspection of the alignments we noted that in almost half of the cases (47% on average) a SNP was originally correctly called as heterozygous by Maq, but excluded from our SNP list in the filtering step. In the remaining cases both alleles were observed by inspecting the alignment manually, but Maq failed to call both of the SNP alleles. Three positions where we obtained homozygous calls both automatically and manually, but were called heterozygous in the HapMap data, were validated by Sanger sequencing. Four additional positions that were homozygous in the HapMap data, but heterozygous in our sequence data and three positions that were homozygous in the HapMap data, but homozygous for the opposite allele in our sequence data were also validated by Sanger sequencing of all three samples. Of the 12 SNPs that were selected for validation, one was not sequenced due to failed PCR. In ten cases, the Sanger sequencing call confirmed our sequencing calls from GAIIx, while in one case the Sanger sequencing call agreed with the HapMap data.

### Known and novel SNPs in the target region

In addition to comparing our SNP calls to the HapMap genotypes, we compared them to all the SNPs that were annotated to our target region in the Ensembl variation database (http://www.ensembl.org). Out of the SNPs that we called, 90–94% of the SNP positions per sample matched positions that were already listed in Ensembl (Table 4). Between 154 and 350 SNPs per sample appeared to be novel. Twelve of the SNPs that were not listed in the Ensemble variation database were randomly selected for validation by Sanger sequencing. Two of the 12 SNPs failed in the PCR step. In eight cases Sanger sequencing confirmed our SNP call. In one case the SNP was correctly called as heterozygous in the SureSelect library, but called homozygous in the Nimblegen library. Manual inspection of the alignment showed that only two of the 40 reads covering this position in the Nimblegen library represented the reference allele. The position was thus called homozygous for the non-reference allele by Maq. One SNP was called heterozygous in the Nimblegen library, but turned out to be homozygous by Sanger sequencing, which could be caused by misalignment of the reads covering this position. The position was not covered in the corresponding SureSelect library. We then compared our SNP lists to the 1000 genomes project pilot 1 release of April 2009 data available at ftp://ftp1.1000genomes.ebi.ac.uk. 91–93% of SNPs per sample matched the SNP positions reported in the 1000 genomes project data. After excluding all known positions from Ensembl and the 1000 genomes project, we identified 67–181 novel SNPs per sample (Table 4 & Supplementary table S2). In the sample NA10860, 55 of the SNPs were identified in both SureSelect and Nimblegen libraries. In the sample NA11992 the corresponding number was 39 and in the sample NA11993 we identified 79 novel SNPs with both methods. Between two and 15 of the novel SNPs per sample annotated to exons, while the remaining novel SNPs were located in introns or in the 3′ or 5′ regions of the targeted genes. Out of the ten SNPs that were successfully sequenced by the Sanger method after excluding all the SNPs that were annotated in Ensembl variation, four SNPs were present in the 1000 genomes data and six were truly novel.

Since the samples that we sequenced here represent two parents (mother NA11993, father NA11992) and a child (NA10860), we could test for Mendelian inheritance of the novel SNPs as quality control to exclude sequencing errors called as SNPs. Using Plink [41], we analyzed 76 novel SNPs called in the SureSelect libraries and 108 novel SNPs called in the Nimblegen libraries that were called in the child and at least one of the parents and were sequenced to at least ten fold coverage in all three samples. There were 17 SNPs (22%) that did not follow Mendelian inheritance when the samples had been prepared using the SureSelect method and 19 SNPs (18%) when the samples had been prepared using the Nimblegen method. These results demonstrate that by sequence capture and sequencing by GAIIx we were able to reliably detect known SNPs in the targeted region and discover new SNPs even in the HapMap samples that have been extensively analyzed previously. Our data further shows that SNP calls obtained from the SureSelect libraries are more reliable, but we can still call a larger number of SNPs correctly using the Nimblegen method.

## Discussion

Thanks to the rapid and continuous development of the new generation of sequencing technologies and simultaneous decrease in sequencing costs, targeted sequencing will likely be used instead of genotyping in future large scale genetic studies. The aim of our study was to determine which sequence enrichment method would be best suited for variant discovery in a region of interest that consisted of continuous genomic intervals.

Specificity and uniformity of the capturing process define how much over-sampling is needed to adequately cover a sequencing library. In our hands, 54% of the sequence reads aligned to the targeted region, when the sequencing libraries were prepared using the Nimblegen method and 74%, when the libraries were prepared using the SureSelect method, on average. Based on these percentages we can calculate that if the library is prepared using the Nimblegen method, approximately 1.4-fold more over-sampling is required to obtain any given average coverage than if the library is prepared using the SureSelect method. We obtained similar uniformity with both methods, which is in agreement with the results of another study that compared the same enrichment methods in exonic regions and conserved elements [42]. Coverage uniformity is important so that one does not need to waist sequencing capacity for over-sequencing of some regions in order to obtain adequate coverage of others. As has been previously shown for hybridization based sequence capture [42], we obtained high uniformity with both methods.

The major difference between our study and the study by Teer et al. that concentrated on exons and conserved regions [42] is that we were able to design Nimblegen probes for 78% and Agilent probes for 55% of our region of interest, whereas Teer et al. were able to design probes for 94% and 92.6% of their region of interest for Nimblegen and Agilent, respectively [42]. Due to this difference in the probe designs between the two methods, we recovered a considerably (0.6 Mb) larger proportion of the original target region from the Nimblegen libraries. To define repeats, Agilent uses RepeatMasker. RepeatMasker defines repeats according to different criteria than the in-house algorithm that Nimblegen uses, which explains the differences in the probe targeted regions between the two methods. We could recover a slightly larger proportion of the probe targeted regions from the SureSelect libraries and a slightly larger proportion of the non-repeated part of the targeted region from the Nimblegen libraries. This was surprising since Agilent used RepeatMasker to exclude repeats from the SureSelect design and we also used RepeatMasker to define the repeats when we analyzed the non-repeated portion of our target region. It is likely that the regions, which are included in the Nimblegen design, but excluded from the SureSelect design, are more difficult to capture. It would be interesting to compare the two methods employing the same repeat masking strategy for both to see how that would influence the results. It is possible to submit an already RepeatMasked region to Nimblegen for a design and a custom repeat masking option is currently available when ordering SureSelect capture probes, but it is dependent on extra bioinformatics work by the customer. Better coverage of the whole target region will be increasingly important as the sequencing read length gets longer and reads can be aligned to more repeated regions.

We called more known and novel SNPs from the Nimblegen libraries than from the SureSelect libraries in each sample, which is expected since we also sequenced a larger proportion of the target region from those libraries. We observed slightly better concordance between our SNP calls and the HapMap genotypes from the SureSelect libraries, which is probably due to the larger percentage of reads mapping to the targeted region and the targeted region being smaller in the SureSelect libraries. These two factors combined lead to higher average coverage in the SureSelect libraries despite the downsampling of the number of sequence reads in the SureSelect libraries to equal those in the Nimblegen libraries in the beginning of the analysis. Our SNP calls from both types of sequencing libraries correlated very well with the HapMap genotypes on the individual level reflecting the high quality of the SNP calls. Ten out of the 11 discrepant calls that were investigated by Sanger sequencing further agreed with our sequence data. Our SNP calls also correlated very well with the data in Ensembl variation as well as with the SNP calls from the 1000 genomes project. In addition to being genotyped by the HapMap project, the parents of the trio that we sequenced here are part of the 1000 genomes pilot one cohort of 180 unrelated individuals that have been sequenced to low coverage. This explains why we find so few novel SNPs in our samples. Nevertheless, we do find some new SNPs highlighting the importance of high coverage sequencing to detect all the variation in a particular region.

Particularly when a large number of samples are to be sequenced, practical considerations are important in the choice of the sequence enrichment method. The SureSelect hybrid capture protocol is faster and easier to perform than the Nimblegen protocol, which is a general difference between array and solution based capture methods rather than between these two methods in particular. Another advantage of liquid-based capture methods is that they are easier to multiplex and automate than array based ones. Multiplexing and automation will become more important in the future, especially when targeted sequencing is being used instead of genotyping in studies involving a large number of samples. Solution based capture methods also require smaller amounts of input DNA, which is especially important when clinical samples are studied.

Taken together, our results show that the strengths of the SureSelect method are better specificity and reproducibility, and the strength of the Nimblegen method is better overall coverage of the original target region, which also results in a larger number of SNP calls per sample. Thus Nimblegen's method performed better in discovering variants in our target region. We conclude that repeat masking and probe design are important parameters when one wants to capture all the variation in a given region. Combining the strengths of both methods should be useful in the further development of sequence capture methods for massively parallel sequencing in the future.

## Supporting Information

Table S1 Primer sequences for Sanger sequencing.
(DOC)

Table S2 Novel SNPs.
(XLS)
