# Supplementary material for: Performance of Microarray and Liquid Based Capture Methods for Target Enrichment for Massively Parallel Sequencing and SNP Discovery
Source: PLoS One. 2011 Feb 9;6(2):e16486. doi: 10.1371/journal.pone.0016486 (PMC3036585; doi:10.1371/journal.pone.0016486)
Supplement: Table S1 — Primer sequences for Sanger sequencing. (DOC) [file pone.16486.s001.doc]

**Supplementary Table 1.** Primer sequences

| Variant coordinate (build 36) | Forward primer sequence | Reverse primer sequence |
| --- | --- | --- |
| chr15:23506522 | CCCTATGGTGACAGGAGGAA | GTGGGCACTGGAGTCATCTT |
| chr15:23582491 | GAGAGCACCAGGGAGAAGTG | gcatgaaatgtgcttgagtga |
| chr14:51566495 | AAAAACGCCCAAAACATCTG | tgtgccacttcctgactttg |
| chr14:51570537 | agccagtgcctgagtttgag | CTCCCACCCCAGTCAAATAA |
| chr14:51570579 | ccaccattgggtagaggtca | ACTGGCAGGTACCAAGTTGC |
| chr14:51570703 | TGGTACCTGCCAGTTTGCTT | TGTTGGGTGTAGGCATCTGA |
| chr7:158341766 | ggcctcaaacgtaaagctct | GGGTAGGTGCAGTACCCTGA |
| chr7:158400500 | ATTTCGCCAGCTGTCTCATC | tcaaccagcgtgttgacatt |
| chr7:158402700 | TGAAATGTTGACACGTGGTT | GGTGTTTAGTGACGTCTCACAAT |
| chr7:158402762 | ATTGTGAAATGTTGACACGCT | GAACGGGCAAGAAAaggagt |
| chr19:62696743 | gctttcaggcataattccaca | ccagtatgaacacgccaatg |
| chr16:3705036 | GCACATGCTACAACCTGGAA | GTGCGGTGGTTCAAGCATTC |
| chr5:1152688 | CTCAGCGGGTCAGAGGATT | TCCCGAGTCAGCCTTTCTAA |
| chr22:17656340 | TGAAACTGCGAAAACACTCG | CCTCCTGAGGGTCCTTTCTC |
| chr22:21977800 | ACCATGCAAAGTGGTCAGTG | GCAGGAGCCTTGAAAGAATG |
| chr17:1612958 | TTAGGCCGGCAGGTGTATAG | AGCAGCCCCAAGAATAGGAC |
| chr16:88327664 | AGCTGTCAGTGTGAACCTGG | TGCTCCTTGATGTGCTTCTG |
| chr17:74348631 | GCCTCACACACGTGTCACC | CGCCACTGTCACACTTCAG |
| chr16:2014224 | CCCTTTCTTCGAGACACCAA | GAGAGGGGACAGTGAGTCCA |
| chr5:1165994 | AGGCAACTCCAGTGGAAGGA | TTCTGGAACATTTCCCAAGG |
| chr17:7774314 | gacggtccccacaataaaga | TTATCCCCTGCCTAATGCTG |
